# Supplementary figures and images for: Health Characteristics of Patients with Cystic Fibrosis whose Genotype Includes a Variant of the Nucleotide Sequence c.3140-16T>A and Functional Analysis of this Variant
Source: Genes (Basel). 2021 May 28;12(6):837. doi: 10.3390/genes12060837 (PMC8229552; doi:10.3390/genes12060837)

**Patient's intestinal biopsy**

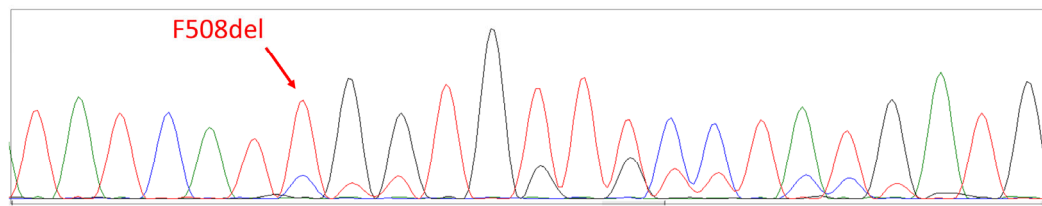

**Patient's nasal epithelium**

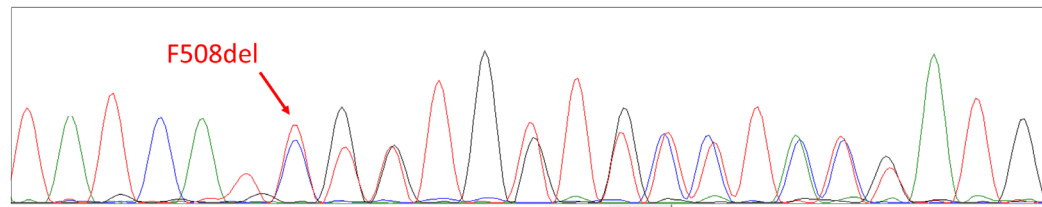

Supplement: Supplementary file 1 [file genes-12-00837-s001.zip › genes-1213332-supplementary.pdf]
